# Supplementary material for: Learning curve and surgical outcome of robotic assisted colorectal surgery with ERAS program
Source: Sci Rep. 2022 Nov 29;12:20566. doi: 10.1038/s41598-022-24665-w (PMC9709162; doi:10.1038/s41598-022-24665-w)
Supplement: Supplementary file 2 — Supplementary Information 2. [file 41598_2022_24665_MOESM2_ESM.zip › RAL Raw data-Ñ[▒K/072/OP N_ñw╝╨░O▒Kñσ.pdf]

W92 / W92

# 臺中榮民總醫院
## 手術室護理記錄

[redacted]

### 基本資料

- 室別： 開刀房-17室，手術科別： CRS，手術類別： 常規，日期： 2021/04/08
- 進入時間： 0750 ； 麻醉開始時間： 0755 ； Time Out時間： 0945 ； 劃刀時間： 0946 ； 手術結束時間： 1545 送出時間： 1600 ；
- 麻醉方式： GE

  診斷： Malignant tumor of rectum

  手術名稱： 1. Robotic (XI) assisted low anterior resection
  2. Intraoperative colonoscopy : (-).
  3. High ligation (-) : IMA was not approached.
  4. Take down from the splenic flexure : (-)
  5. Robotic EndoGIA (45-4.3) X2 + EEA (33mm) were applied to complete the end-to-end anastomosis
  6. Proximal cut end: 5 cm, distal cut end: 2 cm
  7. A 12 Fr. penrose drainage tube was inserted from RLQ of abdomen into the pelvic
  8. ADHESION BARRIER FILM was placed below the centrol extended wound

  手術局部用藥： NIL

  術前預防性抗生素：
    - 抗生素名稱一： Veterin 2GM IVD/由麻醉科護理人員給予
    - 時間： 0910
    - 上次簽章: 陳家瑋

  ，追加預防性抗生素：
- 手術醫師： 蔣鋒帆，林陳彥，楊曙榮，陳威遠，陳琬清
- 麻醉醫師： VS 吳志成
- 
    - 刷手護士A ： 林怡廷，時間： 0750，刷手護士B ： 陳盈妤，時間： 0750，接替刷手一： 陳盈妤，時間： 1100，接替刷手二： 林怡廷，時間： 1130
    - 流動護士A ： 陳家瑋，時間： 0750，接替流動一： 周筱涵，時間： 1230
- 更多接替及流動護士： 接替刷手三： 陳慧瑜，時間： 1230

---

### 皮膚狀況

A-V Shunt： 無

術前： 完整

記錄者： 上次簽章: 陳家瑋

術後： 不完整

部位(術後)：

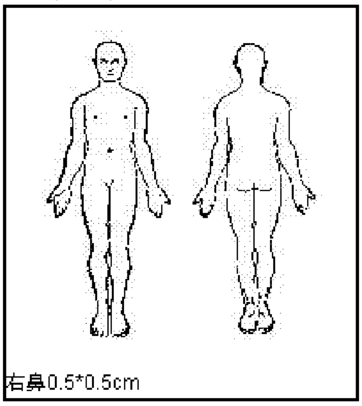


記錄者： 上次簽章: 周筱涵

---

### 手術準備

手術臥式 單一手術臥式

- 
    - 手術臥式： 截石臥式
    - 使用附件： 溫毯 38 ℃，頭圈，腳架，其它： 海棉、silicon pad*2、側身架、頭架

- ○ 皮膚準備： 10%Aq Betadine，Hibiscrub，2% Chlorhexidine
- ○ 電燒： 使用電燒: 電極片位置： 右大腿，雙極電燒，記錄者： 上次簽章: 陳家瑋

止血帶T： 無

## 植入物

- 植入物名稱/號碼/數量/部位： DAVINCI Xi EndoWrist Stapler 45 Green Reload*2/rectum

, 記錄者： 上次簽章: 周筱涵

## 標本送檢

- 細菌培養 無
- 細胞學 無
- 冰凍切片 無
- 病理標本
  組織名稱一：rectum
  ,
  組織名稱二：distal cut end
- 截肢 無

標本交付 標本： 2 袋，標本： 0 盒，標本： 0 瓶，細胞學： 0 管，Culture Tube： 0 管，被交付醫師： R 陳威遠，

- 記錄者：

## 傷口術後敷料

傷口敷料： 傷口敷料： 無

- 請於圖上標示部位：

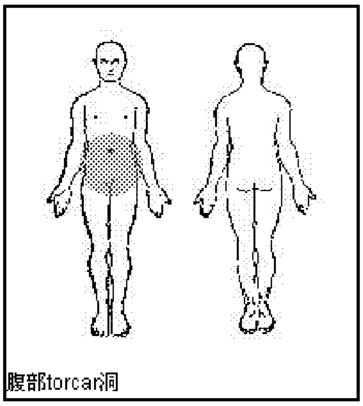


- 傷口敷料：軟性敷料。

## 導管

無 導管資料

## 用物計數

- 手術中出血量： minimal c.c.
- 手術中尿量： 300 c.c.
-
  - ○ 其它： 無

用物計數： 多切口同時縫合

- 1.器械： 正確，2.縫針： 正確，3.刀片： 正確，4.紗布： 正確，5.腹部墊： 未使用，6.紗球： 未使用，7.PEANUTS ： 未使用，8.紗條： 未使用，9.絲帶： 未使用，10.針頭： 未使用，11.Vessel loop： 未使用，12.Boots： 未使用，13.Jelco： 未使用，14.棉條： 未使用，
  - ○ 第一次：
    - ■ 刷手護士 ： 林怡廷
    - ■ 流動護士： 上次簽章: 周筱涵
    - ■ 時間： 1425

  ,
  - ○ 第二次：
    - ■ 刷手護士 ： 林怡廷
    - ■ 流動護士： 上次簽章: 周筱涵

- 時間： 1445

,

- 第三次：
  - 刷手護士 ： 林怡廷
  - 流動護士： 上次簽章: 周筱涵
  - 時間： 1455

## 備註

備註： 達文西 0度 30度 scope NO.2
R6 腹腔鏡 NO.2

- 記錄者： 上次簽章: 陳家瑋
- 時間： 0810

填寫護理人員 陳家瑋

正式摘要

W92 / W92

doctorRank:B
